# Asymptomatic Infant Rib Fractures Are Primarily Non-abuse-Related and Should Not Be Used to Assess Physical Child Abuse

**DOI:** 10.3390/children10111827

**Published:** 2023-11-20

**Authors:** Martin J. C. van Gemert, Marianne Vlaming, Steven C. Gabaeff, Peter G. J. Nikkels, H. A. Martino Neumann

**Affiliations:** 1 Department of Biomedical Engineering & Physics, Amsterdam University Medical Centers, Location AMC, 1105 AZ Amsterdam, The Netherlands; 2 Private Practice, Criminal Psychology and Law, 6986 CL Angerlo, The Netherlands; marianne@vlamingadvies.com; 3 Clinical Forensic Medicine, Healdsburg, CA 95448, USA; stevengabaeff@gmail.com; 4 Department of Pathology, Wilhelmina Children’s Hospital, University Medical Center, 3584 CX Utrecht, The Netherlands; p.g.j.nikkels@umcutrecht.nl; 5 ZBC-Multicare, 1217 AB Hilversum, The Netherlands; martino.neumann@gmail.com

**Keywords:** child abuse, infant resuscitation, asymptomatic rib fractures, rickets, birth trauma, prematurity, osteogenesis imperfecta, hypermobile Ehlers-Danlos-Syndrome, chronic histiocytic intervillositis, vitamin-D deficiency, false allegations, misdiagnosis

## Abstract

Finding infant rib fractures was for many years an almost undisputed proof that physical child abuse took place. Yet, these rib fractures are virtually always occult and asymptomatic and are only identified when looked for, usually with X-rays, from physical child abuse accusations related to, e.g., suspicion of the shaken baby syndrome. In a recent systematic literature review (searched in Cochran, Embase, PubMed and Sociological Abstracts), Güvensel questioned the diagnostic accuracy of rib fractures to be caused by abuse, due to lack of sufficient scientific evidence. Further, there is currently a world-wide disagreement between physicians considering themselves child abuse specialized, and physicians that explore non-abuse-related symptoms that may mimic physical abuse, which, it is hoped, will significantly reduce current unjustified child abuse diagnoses. In an attempt to help resolving this disagreement, we hypothesize that the probability of physical child abuse-related infant rib fractures is significantly lower than the probability of all other possible non-abuse-related causes of occult asymptomatic infant rib fractures, e.g., from birth trauma, prematurity, osteogenesis imperfecta, hypermobile Ehlers-Danlos Syndrome, severe chronic placental pathology (e.g., massive perivillous fibrin depositions and severe chronic histiocytic intervillositis), and vitamin-D deficiency. As method, we attempted to assess the incidence of these various causes of infant rib fractures, in the Netherlands and the USA. The results are that the estimated Dutch and USA physical abuse-related infant rib fracture incidences are at least about 250 and 45 times lower than the sum of all the non-abuse-related estimates. Because these latter rib fractures are occult and asymptomatic, it is likely that (many) more could be out there. In conclusion, occult asymptomatic rib fractures develop perinatally, virtually always as birth trauma, in infants with sufficiently weak bones due to vitamin D deficiency, transmitted by their vitamin D deficient pregnant mothers. This group also includes cortical rib cracks due to deformation forces, with an estimated 186/100,000 incidence. And, despite obvious uncertainties in all estimated incidences, we provided strong evidence that our hypothesis has relevance, implying that the abundant occult asymptomatic rib fractures, when found in infants, should not be used to assess potential physical child abuse.

## 1. Introduction

Infant rib fractures are well-known to exist albeit that they are commonly without clinical signs, and their possible causes are so far incompletely clarified. Their incidence is still unknown. These asymptomatic fractures were only identified when looked for, usually with X-rays, based on physical child abuse accusations, e.g., related to the presence of bruises or suspicion of the shaken baby syndrome. Physical child abuse related rib fractures will be discussed below in Section 3.1 and Section 4, paragraph 4. For many years, the identification of such occult asymptomatic rib fractures in infants was frequently an undisputed sign that physical child abuse had taken place. However, it was ignored that occult asymptomatic rib fractures occur frequently in infants, e.g., [1,2,3]. It is therefore essential to be able to distinguish between different causes of infant rib fractures. Only then will non-abuse-related symptoms that may mimic physical child abuse become easier identified and, hopefully, will significantly reduce unjustified child abuse diagnoses for an improved safety of innocent families.

In Güvensel’s thesis [4], a systematic literature review was described, aimed to answer the question “*With what certainty can it be claimed that rib fractures (or classic metaphyseal lesions) in infants are attributed to physical abuse?*”. The search (with Cochrane, Embase, PubMed and Sociological Abstracts) identified 4512 abstracts and 35 articles of which 426 papers were analyzed on rib fractures as a sign of physical child abuse, e.g., [5,6,7]. It was for example indicated that the Barsness study [5], stating a strong association of rib fractures with non-accidental and severe trauma, with a 95% positive predictive value, is representative for how studies of child abuse are conducted with flawed methodology, i.e., with a high risk of incorporation bias, mainly circular reasoning and misconception. Güvencel [4] concluded “*there is insufficient scientific evidence that rib fractures can be caused by abuse and therefore also insufficient scientific evidence on which to determine the diagnostic accuracy of rib fractures to be caused by abuse*”.

Further, there is currently a serious world-wide disagreement between physicians considering themselves child abuse specialized, and physicians with protracted and profound interest in child abuse practices and legal injustice, exploring alternative, non-abuse-related mechanisms, which produce symptoms that may mimic those from physical abuse. Symbolic for the former group is Brown et al. [8], representing the (American) “*Society for Pediatric Radiology Child Abuse Committee*”, and for the latter group Miller et al. [9]. Brown et al. [8] stated that it was “*grossly irresponsible*” and could present a “*grave public health risk*” that Miller et al. [9] reported their findings on genetic causes of bone fragility and fractures. Also, Brown et al. referred to Strause [10], who invented the term (child abuse) “*denialists*”, as “*an example of the evidence based medical literature*”. Neither Brown nor Strause referenced publications showing that genetic affected infants, e.g., suffering from osteogenesis imperfecta and Hypermobility Spectrum/EDS Syndrome, have symptoms that are frequently misdiagnosed as child abuse [11,12,13,14], and neither to the Swedish population-based registry studies of 1,855,267 infants by Högberg et al., e.g., [3,15].

In an attempt to help resolving this disagreement between establishment thinking and challengers’ reasoning, we aim to address the following hypothesis:

“*We suggest that the incidence of infant rib fractures caused by physical child abuse is significantly lower than the incidence of rib fractures resulting from various other non-abuse causes.*”

These non-abuse causes refer in this paper to birth trauma, prematurity, osteogenesis imperfecta (OI), hypermobile Ehlers-Danlos Syndrome (hEDS), massive perivillous fibrin depositions and severe chronic histiocytic intervillositis and, most commonly, fetal-neonatal bone weakness from a widespread vitamin D (vitD) deficiency as transmitted by vitD deficient pregnant mothers to their newborns.

## 2. Method

We will attempt to assess the incidence of the various causes of infantile rib fractures, in the Netherlands and the USA. We restricted ourselves to these 2 countries because of the obvious importance for the authors, from the Netherlands and the USA, and also the advantages of the yearly available child abuse incidences of the Children’s Bureau of the U.S. Department of Health and Human Services (https://www.acf.hhs.gov/cb/data-research/child-maltreatment, accessed on 1 September 2023). We scanned publications referring to each of the non-abuse mechanisms mentioned in the previous paragraph, to estimate their incidences. The rib fracture incidence from the relative number of rib fracture cases could be estimated in the mechanisms described in Section 3.1, Section 3.2, Section 3.3, Section 3.4 and Section 3.7.2. In the remaining Section 3.5, Section 3.6 and Section 3.7.1, rib fracture details were not given and a hypothesized estimate was made by the percentage that each of these clinical mechanisms could be linked with osteogenesis imperfecta, which rib fracture incidence was given (Section 3.4).

## 3. Results

Table 1 summarizes the estimated incidences of the 8 mechanisms that we have calculated below.

### 3.1. Physical Child Abuse

The incidence of infant rib fractures caused by physical child abuse is unknown. We hypothesized that a reasonable guess can be obtained by combining known physical child abuse incidences with the estimated rib fracture incidence originating from resuscitation procedures in infants that results in extreme deformations. We will use an estimated physical abuse incidence in Dutch children < 1 year of age, based on available data from Belgium [16], the Dutch southern neighbor country. In the Appendix A, we derive that the Belgian physical abuse incidence of the first 3 years is about 0.00064, which we will use as the current next best Dutch physical abuse prevalence for infants.

The incidence of rib fractures following resuscitation procedures was estimated from published reports. i.e., in 2 out of an estimated 741 infants from 6 papers mentioned in Table 2 of Maguire et al. [17], i.e., [18,19,20,21,22]; 0 in 50 neonates [23]; 19 in 571 autopsies in infants < 6 months [24]; 8 in 70 [25]; 0 in 50 [26]; 0 in 6 [27]; and 24 in 546 sudden unexpected deaths in infancy [28]. An estimated incidence then is (2 + 0 + 19 + 8 + 0 + 0 + 24)/(741 + 50 + 571 + 70 + 50 + 6 + 546) = 53/2034 ≈ 0.026. Our hypothesis thus is that the incidence of Dutch infant rib fractures due to violent physical abuse is most likely less than 0.00064 × 0.026 = 1.7/100,000 children.

For USA children < 1 year of age in 2016, the physical abuse incidence was ≈ 4.51/1000 children [29], thus the estimated incidence of rib fractures is less than 0.00451 × 0.026 < 12/100,000 children.

Rib fractures from violent causes, here physical abuse and resuscitation, can be identified relatively easily during the first few weeks after injury by pain symptoms that the child experiences during normal handling (SCG, personal experience).

### 3.2. Birth Trauma

Abedzadeh-Kalahroudi et al. [30] found 1 visible rib fracture in 7154 live births during 2012 and 2013 in Kashan, Iran. Högberg et al. [31] described 12 rib fractures found in 1,855,267 children under 1 year of age. Of these 12 cases, 2 were from accidents, 2 occurred in preterm children, and 3 were in small-for-gestational-age children (<2500 g birth weight). Five rib fractures were found in >4 kg weighing neonates, and we assumed that birth trauma developed in these babies. Thus, 5 in 1,855,267 neonates. In 1964, Rubin [32] found in 15,435 successive deliveries that 108 newborns had birth injuries visible on imaging, however, none had visible rib fractures. In [33], zero rib fractures were found in 2 studies, a Dutch registry study of 158,035 hospitalized full-term neonates (reference 23 of [33]) and from 115,756 births in 5 studies (references 24–28 of [33]). All these studies disregarded the more common deformation-induced occult rib cracks, with no displacement, that are sufficient to produce healing callus formation, which can be seen only in the weeks and months after the labor contraction-induced cracks to the ribs have occurred. We can thus use thru and thru rib fractures, those that be used to identify abuse, in heavy weighing fetuses, in the USA and the Netherlands, and estimate an incidence of about (1 + 5 + 0 + 0 + 0 + 0)/(7154 + 1,855,267 + 15,435 + 158,035 + 115,756) = 6/2,151,647≈ 0.3/100,000 births. Occult rib cracks from abuse can occur, but are identified only if symptomatic. Such cracks without significant soft tissue injury and pain symptoms are almost never identified due to lack of collateral injury. The almost always occult nature of cracks, abusive or not, limits identification.

### 3.3. Prematurity (Birth before 37 Weeks of Gestation)

Amir et al. 1988 [34] identified 973 premature babies of which 7 had visible rib fractures. Lucas-Herald [35] found 26 of 1446 < 37 weeks premature babies with rib fractures visible on imaging. Thus, an estimated rib fracture incidence in <37 weeks premature babies of (7 + 26)/(973 + 1446) ≈ 0.014. In 2015 in the Netherlands [36], it was determined that 5.3% of all births in 2015 were premature babies. The Dutch incidence of premature-related visible rib fractures could then possibly be (5.3/100) × (14/1000) ≈ 74/100,000 children.

The USA prematurity incidence in 2021 [37] was 10.5%, thus the estimated USA incidence of rib fractures from prematurity was about (10.5/100) × (14/1000) ≈ 147/100,000.

Prematurity further impacts on occult rib fractures is due to the decreased calcification in fetal bones [38]. This is in addition to decreased calcium in bone due to vitD deficiency. Each week of additional prematurity (<37 gestational weeks) results in ≈ 6% decrease in total body calcium down to 25 gestational weeks, and about 80% decrease in body calcium level. Thus, the 47/100,000 and 147/100,000 incidences could well be an underestimation of the true incidences.

### 3.4. Osteogenesis Imperfecta (OI)

Osteogenesis Imperfecta (OI) has a reported incidence between 1 in 10,000–20,000, say 1:15,000. From [39], ≈15/78 ≈ 19% OI-children had rib fractures, thus ≈1 in 15,000/0.19 ≈1 in 78,000 OI children, or an OI-related estimated rib fracture incidence of ≈1.3/100,000, in the Netherlands and the USA.

### 3.5. Hypermobile Ehlers-Danlos-Syndrome (hEDS)

Table 3 of Miller [40] gives 7 classes of infant risk for bone fragility when the infant has 1 or 2 hEDS parents. In 2 of these 7 classes, the mother has hEDS, giving maximal risk for infant bone fragility.

We hypothesized that in such cases of maximal bone fragility, the hEDS-related infant has the same probability of acquiring rib fractures as OI infants. So, the estimated rib fracture incidence of infants from an hEDS-mother is the product of hEDS-incidence and the 19% probability of OI-infant rib fractures from [39]. Using an hEDS incidence of about 1 in 5000 [41], the estimated infant rib fracture incidence is about (1/5000) × 0.19 ≈ 4/100,000 children.

### 3.6. Chronic Placental Histiocytic Intervillositis

If the placenta is malfunctioning, for example in cases of a serious chronic histiocytic intervillositis with a massive perivillous fibrin deposition [42,43,44], the reported incidences are (1), in 6 of 10,000 2nd and 3rd trimester placentas [42] versus (2), in 20 diagnoses of 22,000 births over a 6 years period [44], or 20/22,000 ≈ 9.1/10,000 births. An average estimated incidence then follows as (6 + 20)/(10,000 + 22,000) ≈ 8.1/10,000 births. Fragile bones likely develop, but rib fractures have been described rarely, we found 2 published cases [43,44]. A possible estimate of rib fracture incidence can be made by using that about 78% live births occur [45] of which 13 in a study of 36 cases were born at term, or ≈ 36% (Bos et al., 2023 [46]). We assumed that only severe cases of chronic histiocytic intervillositis may develop bone fragility comparable to osteogenesis imperfecta; these were reported in 17% of the cases [46], stating that only intervillositis but not villitis and perivillous fibrin depositions correlated with severity. Then, an estimate rib fracture incidence is about (8.1/10,000) × 0.78 × 0.36 × 0.17 × 0.19 ≈ 1/100,000 infants.

### 3.7. Vitamin-D Deficiency

Vitamin D (vitD) serum concentrations are expressed in ng/mL as well as in nmol/L. Their relationship is that 0.4 ng/mL is equivalent to 1 nmol/L, e.g., 50 ng/mL equals 125 nmol/L.

Normal has been defined for decades as >30 ng/mL (>75 nmol/L), insufficient as 20–30 ng/mL (50–75 nmol/L), and deficient as <20 ng/mL (<50 nmol/L, see also [47]. However, in 2022, the American Academy of Pediatrics changed the values for vitD deficiency to <15 ng/mL (<37.5 nmol/L) and sufficiency to >20 ng/mL (>50 nmol/L), thus insufficiency is between 15 and 20 ng/mL (37.5 and 50 nmol/L). This change implies that visible bony abnormalities, e.g., rib fractures, now occur more easily at “new” normal considered conditions, which favors physical abuse suspicions. Gordon et al. 2008 [48] found extreme vitD deficiencies of <8 ng/mL in 4 of 247 healthy infants (fraction Fr ≈ 1.6%), 26 had <20 ng/mL (Fr ≈ 10.5%), and <30 ng/mL occurred in 97 children (Fr ≈ 39.3%). The excel-derived trendline gave:

vitD(ng/mL) = 6.9024 × ln(Fr) + 36.128(1)

In another study [49], slightly higher rates were found.

**Figure 1:**
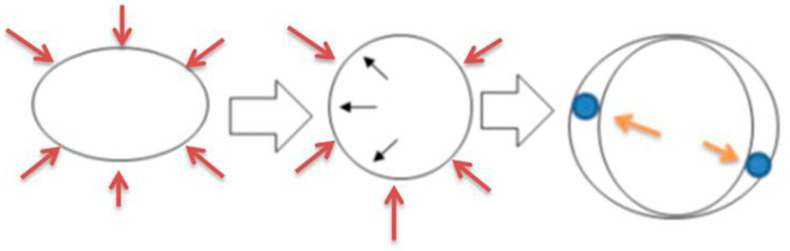
(Figure 5 of Gabaeff [50], with permission from the author). The substantial forces during uterine contractions are applied equally all around (red arrows in the far left side of the graphic) to the rib cage which is normally oval in shape. This transforms the oval to a more circular shape and stresses the inner cortex of the boney ribs (middle configuration) and, if deformed enough, to a breaking point where distraction cracks or overt fractures can occur. With vitD deficiency and infantile rickets (vitD/IR), and an osteoporotic state, the probability of damage and cracks increases. The ribs are much more prone to extreme deformation cracks and fractures to occur at less than the normal force required to break ribs in healthy bones. Because the bones are diseased they are called “pathologic fractures” (fractures in diseased bones). In the far right configuration, the small blue circles are the baby’s arms. The arm’s location is variable (anterior, lateral, and posterior) in the uterus and during labor they move around. Where they are during a contraction is where deformation and stress forces are highest. The movement of the arms accounts for unpredictable locations of cracks, fractures, and callus seen weeks later on skeletal survey. Thus, rib callus positioning/location, misused to allege abuse, has no probative value (in spite of statements from child abuse pediatricians that they do), in diagnosing abuse vs. vitD/IR causation based osteoporosis caused by vitD deficiency and infantile rickets (vitD/IR).

Fetal vitD deficiency can cause bone weakness, structural abnormalities and fetal rickets, which continue in the newborn, and are visible on imaging and at autopsy, e.g., [49,50]. In Figure 1, which reproduces Figure 5 from Gabaeff [50], we are showing the mechanisms that demonstrate the deformation forces applied to the ribs and rib cage during labor contractions. However, regarding resolution of fetal and newborn rickets, bone strength improves during the first 6 months of age about 3-fold compared to the level at birth [40]. Furthermore, specific findings related to the resolution process may sometimes be mischaracterized as other and older injuries entailing a risk for augmentation of an existing false abuse narrative and to buttress legal prosecutions of likely innocent caregivers. Ziegler et al. [51] studied the effects of vitD supplements in 1 month-old breastfed infants and showed 12–15 ng/mL average vitD improvement at 2 months in the 4 supplement doses (200, 400, 600 and 800 International Units (IU) per day, where 40 IU = 1 μg). At 9 months, at the end of the study, the average vitD improvements across the spectrum of resolution protocols, were 2.7–3.7 ng/mL per month. However, Płudowski et al. [52] provided Guidelines for Preventing and Treating Vitamin D Deficiency, from infants to adults, and recommended a supplement dose of 1000 IU in infants, versus 3000–5000 IU for 2–18 year-old children. More rapid increasing vitD levels are beneficial.

In the USA, fetal vitD assessment, e.g., through blood sampling of the mother, is almost never done, not by obstetricians and nor in newborns by pediatricians (Michael G. Ross, personal communication to MJCvG), even when bony findings are central to prosecutions of abuse. Because there is a clear need to do so, one of us (SCG) is attempting to start an obstetric initiative to check maternal vitD levels at the beginning and during pregnancy, which will likely decrease false accusations of child abuse after birth [50]. In the Netherlands, most neonates that are admitted to the Neonatal Intensive Care Unit have low vitamin-D levels. All have a degree of neonatal rickets proportional to their deficiency level. Mild, moderate and severe rickets can be distinguished by routine X-ray investigations; treatment is e.g., by high dose supplements that accelerate increasing the baby’s vitD levels, augmented by parenteral calcium over weeks to months.

As expected from literature, vitD deficiency has a reasonably large incidence in pregnant woman. Keller and Barnes [53] reported that 18% of UK pregnant women in 2008 had a very serious <10 ng/mL vitD deficiency. The combination of low maternal vitD and the resistance of vitD molecules to penetrate through the maternal-fetal blood barrier, contribute to the low values in their offspring, estimated as about 80% of the maternal value (Table 2 of [49]). Also identified in [49] was that 96% of random birthing mothers in Pittsburg were below normal baseline at birth (5% of white mothers were deficient, <15 ng/mL, and 42.1% insufficient, 15–32 ng/mL; 29.2% of black mothers were deficient and 54.1% insufficient). Thus, the number of vitamin-D deficient mothers in the USA is roughly 60–90% of the approximately 4 million that will give birth this year and about 80% of their healthy newborns will have subnormal vitD levels in their cord blood; whites: 9.7% deficient, 56.4% insufficient; blacks: 45.6% deficient, 46.8% insufficient (Table 2 of [49], and see also [54]).

It is known that vitamin-D deficiency in fetuses is responsible for rickets and, with it, perinatal deformation fractures, including rib fractures and other visible bony abnormalities [50]. Keller and Barnes 2008 [53] described 4 cases of term 2–4 months old newborns (3 were 2 months old), all with 1 or more fractures, and 2 (the 1st and 4th cases) with rib fractures. The 1st pregnant mother had 8.7 ng/mL vitD concentration, the 2nd < 4 ng/mL, the 3rd had 14 ng/mL, but the value of the 4th, Afro-American mother was not determined. These cases were sent from different health institutions in the USA to Keller and Barnes for undergoing evaluations for child abuse (Patrick D. Barnes, personal communication to MJCvG). This appears to be the tip of an iceberg of significant bony abnormalities that are misdiagnosed as abusive injuries. Rib fractures, seen as healing callus during a large percentage of skeletal surveys during child abuse workups done in the first year of life, are quite common, see the references mentioned in the Introduction as well as those in previous work [55].

#### 3.7.1. Attempted Prediction of vitD Deficiency-Related Rib Fracture Incidence

As with the placental histiocytic intervillositis complication (paragraph 3.6), a possible estimate of rib fracture incidence can be made by first assuming that serious fetal vitD deficiency in the deficiency range of, say, <20 ng/mL, will develop bone weaknesses and can cause rib fractures. Thus, we assumed that about 10.5% of the newborns are at risk, estimated from the excel-derived trendline of the data from [48], Section 3.7, Equation (1). We further assumed that also here, 17% of the fetuses will develop bone weakness comparable to OI. The estimated incidence then is about 0.105 × 0.17 × 0.19 ≈ 339/100,000 children.

#### 3.7.2. Attempted Prediction of vitD-Deficiency-Related Nutritional Rickets Incidence

The physical manifestations of rickets have not (yet?) been identified in a large study, however, based on personal experience (SCG), it is expected at levels of <24 ng/mL. Then, from Gordon et al. [48], about 17.5% of healthy newborns are at risk, again estimated from the excel-derived trendline of Equation (1) from the data Gordon et al. [48]. Najada et al., 2004 [56] included 443 hospitalized children between birth and 2 years of age and identified 47 (10.6%) with nutritional rickets. Thus, and assuming that the 10.6% incidence also holds for the infants, an estimated incidence is about 0.175 × 0.106 ≈ 0.0186 or about 186/100,000 infants. In the USA, with almost 4 million yearly births, thus equates to ≈74,000 young children per year.

## 4. Discussion

In the Netherlands, the visible physical abuse-related estimated rib fracture incidence is ≈<1.7/100,000, versus the sum of rib fracture incidences due to other causes (birth trauma, prematurity, OI, hEDS, chronic histiocytic intervillositis, and newborns from vitamin-D deficient mothers), is ≈0.3 + 74 + 1.3 + 4 + 1 + 339+ ≈ 420 per 100,000 children, so much more frequent (about 420/1.7 ≈ 250 times) than caused by physical abuse. For the USA this is ≈12/100,000 infants from physical abuse, versus (0.3 + 174 + 1.3 + 4 + 1 + 339)/100,000 ≈ 520/100,000 from the alternative causes, so here a factor of about 520/12 ≈ 45 times more frequent. Summarizing, Dutch and USA infant rib fractures occur about 250 and 45 times less frequent from abuse than from alternative causes. Our incidence estimates suggest that, except for birth trauma in larger newborns, chronic placental histiocytic intervillositis and OI, which have incidences somewhat smaller than that of physical abuse, all other non-abuse-related rib fracture mechanisms occur equally frequent or more often than physical abuse, and that prematurity contributes significantly but that vitD deficiency is by far the dominant mechanism. It would be interesting, and likely productive and rewarding, if this prediction could become further explored, e.g., by checking maternal vitD-levels during pregnancy.

The final estimate of vitD related findings is difficult. Based on clinical experience and the frequency of bony findings in the suspected abuse cases in daily practice, and the findings in [48,53], show that the frequency of vitD deficiency in infants is high. Assuming findings are present at <15 ng/mL and possibly up to 24 ng/mL, many children are impacted by vitD deficiency in a way that can result in false accusations. In the birth statistics of [49], 47.1% of the 52.1% of USA white mothers are deficient or insufficient, and 83.3% of the 15.2% black mothers (incidences from Internet) fall into that range. The number of white mothers is 4,000,000 × 0.521 × 0.471 = 981,564, and the number of black mothers is 4,000,000 × 0.152 × 0.833 = 506,464, thus a total of about 1,500,000 mothers with vitD deficiency sufficient to demonstrate infantile rickets findings in their progeny (from one of us, SCG). We can see that these numbers are a far cry from the statistics gathered via studies looking for obvious thru and thru fractures. Fractures that can be seen on neonatal imaging were highlighted above (Section 3.2 and Section 3.3). The frequency in child abuse cases with mention of bony finding from Susan C. Anthony (personal communication to SCG) is somewhat corroborative at 16.1%, which represents 16.1% of 4 million births, or about 644,000 newborns in the US each year.

A brief comment about the disparity between the low likelihood of physical abuse-related infant rib fracture incidences in the Netherlands (an estimated 250 times less likely than non-abuse causes) and the US (45 times) may be explained by American child abuse pediatric practices that increase the number of so-called “abuse related fractures” diagnosed when similar cases in the Netherlands may be regarded as not abuse related. Overdiagnosis of abuse is likely to occur relatively frequently in the US, dropping the differential from about 250 times less likely than abuse diagnoses in the Netherlands to 45 times less likely in the US, reflecting that abuse is about 5.6 times more likely to be diagnosed in the US. Additional causes may be differences in e.g., health insurance availability, incidence of poverty, living conditions, racism and unemployment, with pediatric child abuse practices as the possible major factor.

It is obvious that the accuracy of the estimated rib fracture incidences is at best very limited. The rib fractures due to premature births is most likely the most accurately assessed outcome whereas that from chronic histiocytic intervillositis is very uncertain. Although the rib fracture incidence from vitD deficiency cannot be very accurate, its large incidence compared to physical child abuse is an accurate outcome. The accuracy of the birth trauma, OI and hEDS predictions are likely somewhat in between. Nevertheless, because infant rib fractures are basically occult and asymptomatic, it can be expected that their incidence can be even (much) larger than we have estimated. As an example, Högberg and Thiblin [3] identified 63 asymptomatic rib fractures in their 1,855,267 collected cases < 1 year of age. Although most of these rib fractures develop perinatally, we acknowledge that a case of OI where rib fractures were identified at 32 weeks in a term pregnancy has also been described [57].

For completeness, we acknowledge that infant rib fractures can exist that are due to physical child abuse, however, without solid proof of their cause, and corroborating clinical symptomatology, the observation as such should not be considered as evidence of a non-accidental cause. Mistakes are family destructive based on accusations alone, and diagnostic accuracy must therefore be paramount, regardless of legal outcome. Also, physical child abuse related rib fractures, as well as when caused by resuscitation, are painful since they are traumatic and forceful (personal observations of SCG). Support for this conclusion can be found in [58], stating that even infant chest physiotherapy and dressing change (wound care) are painful. In contrast, non-abuse-related cases develop from gradual repetitious deformation in undercalcified ribs from labor contractions, virtually almost always perinatally and, without significant soft tissue damage, are hidden and asymptomatic.

The interesting hypothesis that developed during this study is that occult asymptomatic infant rib fractures are (virtually always) birth trauma’s, which developed because of the infant’s weaker bones than normal (thus excluding fall and transport accidents, as well as physical child abuse and resuscitation).

In conclusion we believe to have shown that our hypothesis, suggesting: “*---that the incidence of infant rib fractures caused by physical child abuse is significantly lower than the incidence of rib fractures resulting from various other non-abuse causes*” has scientific significance, which implies that (occult/asymptomatic) infantile rib fractures, when found, should not be used as a proof of physical child abuse. Thus, and despite obvious uncertainties in all estimated incidences, we have hope that our results will not only contribute to the literature on child maltreatment but also to a significant reduction of current unjustified child abuse diagnoses, e.g., [55]. And, also, that innocent caregivers and families will have an increased safety.

## Figures and Tables

**Table 1:** Estimated incidences of the 8 mechanisms as presented in, respectively, Section 3.1, Section 3.2, Section 3.3, Section 3.4, Section 3.5, Section 3.7.1 and Section 3.7.2.

Mechanism	Incidence (per 100,000 Children)	
	Dutch	USA
Physical Child Abuse	1.7	12
Birth Trauma	0.3	0.2
Prematurity	74	147
Osteogenesis Imperfecta	1.3	1.3
hypermobile Ehlers-Danlos-Syndrome	4	4
Histiocytic Intervillositis	1	1
Vitamin-D deficiency	339	339
Rib Cracks	186	186

## Data Availability

The data is contained within the article.

## Appendix A. Estimated Physical Child Abuse Incidences in the Belgian Flanders and Brussels Region

The Belgian child abuse data of 2021 [16] were provided as the number of children reported as victim of child abuse in age-periods of 3 years, in the Belgian region of Flanders and Brussels, which have Dutch as their official language. The first number of victims within 0–2 years of age was 1386, and the first row of Table A1 gives the yearly value of 1386/3 = 462.

The 3 year age-periods of child abused children in Flanders and Brussels were presented in 6 categories: (1): “*Physical neglect or (physical) abuse*” (27.6%); (2): “*Emotional neglect or abuse*” (35.1%); (3); “*Sexual abuse*” (17.7%); (3); “*Inappropriate behavior of the child itself*” (3.5%); and (4): “*Non-responders*” (3.1%). Here, no incidences were provided, however, we retrieved child abuse incidences from the number of abused cases [16] and, from “*Statistics Belgium*” (be.STAT), the number of children <18 year living in Flanders (1,314,555) and Brussels (274,738) in early 2023, thus a total of 1,589,293 children <18 year-of-age in this Belgian region. Per child year, the number of children then is about 1,589,293/18 = 88,294.

We assumed that the fraction of physical abuse within the category “*Physical neglect or physical abuse*” can be derived from the Dutch data [59] that included both “*Physical neglect*” (9 cases) and *“physical abuse*” (7 cases). Thus, the estimated Belgian physical abuse incidences are a fraction of about 7/16 times the “*Physical neglect or physical abuse*” incidence, or a Belgian physical abuse incidence of 0.28 × 0.44 = 0.123.

Total abuse included (Table A1, first row) 462 children in the first year, thus the physical abuse cases are 462 × 0.123 = 57 and a physical abuse incidence of 57/88,294 = 0.00064.

**Table A1:** First row “Belgian Abuse Cases”: based on 1386 cases over 3 years [16], so 1386/3 = 462 cases per year. Second row “Belgian physical child abuse cases”: 12.3% of first-row values. Third row “Belgian Physical Abuse Incidences” (in ‰): dividing 57 by 88,294, the average number of children per child year.

Age (Years)	<1	1	2
Belgian Abuse Cases	462	462	462
Belgian Physical Abuse Cases	57	57	57
Belgian Physical Abuse Incidences (per 1000)	0.64	0.64	0.64
